# Magnetic Nanoparticles for Therapy and Diagnosis in Nanomedicine

**DOI:** 10.3390/pharmaceutics15061663

**Published:** 2023-06-06

**Authors:** Javier Bustamante Mamani, João Paulo Borges, Alexandre Malta Rossi, Lionel Fernel Gamarra

**Affiliations:** 1 Hospital Israelita Albert Einstein, São Paulo 05652-000, Brazil; javier.mamani@einstein.br; 2 Department of Materials Science, Universidade NOVA de Lisboa, 2829-516 Caparica, Portugal; jpb@fct.unl.pt; 3 Department of Condensed Matter, Brazilian Center for Research in Physics, Rio de Janeiro 22290-180, Brazil; rossi@cbpf.br

Magnetic nanoparticles (MNPs) have been widely used for their potential applications, mainly for the diagnosis and/or therapy (theranostic) of several diseases in the field of nanomedicine, as passive contrast agents, through the opsonization process, or active contrast agents, after their functionalization and the subsequent capture of the signal using various techniques such as magnetic resonance imaging (MRI), optical imaging, nuclear imaging, and ultrasound. MNPs are also used as carriers for the delivery of drugs, which can be guided or accumulated through the action of a magnetic field, as well as magnetic biosensors in molecular imaging, magnetic separation, or therapeutic applications, such as the technique magnetic hyperthermia (MHT). These nanoparticles, when functionalized using different therapeutic agents, such as radionuclides, nucleic acids, and antibodies, can be used in combined therapies.

The various configurations of MNPs in the design of structures, chemical compositions, and surfaces enhance their properties, giving the character of multifunctional nanoparticles and thus permitting their simultaneous use in several molecular imaging techniques in a non-invasive way, providing morphofunctional information in different moments, characterization and quantification of biological processes at the cellular and molecular levels, and accurate and timely diagnosis and individualized treatment of diseases (personalized medicine). Technological innovations and the development of new tracers and intelligent probes have further promoted these multimodal imaging techniques, providing better contrast and functional, structural, and morphological information.

This Special Issue of Pharmaceutics highlights advances in the development of MNPs and their applications in the field of diagnosis and therapy in various diseases in in vitro, in vivo, and ex vivo studies.

Of the papers presented in this Special Issue, five papers studied therapies [1,2,3,4,5], three papers aimed at diagnosis [6,7,8], two papers focused on the theranostic process [9,10], and two review articles evaluated the theranostic potential of MNPs in medical applications [11,12].

One of the therapeutic applications of MNPs is their use in the MHT technique, where, due to the MNP heating process, apoptosis of tumor cells is produced when an alternating magnetic field is applied. Therefore, in the publication by Andrade et al. [1], calcium-substituted manganese ferrite coated with citrate MNPs was synthesized using the sol–gel method, and the heating potential was evaluated, proving this technique to be viable for future application in MHT. The publication by Mamani et al. [4] aimed to evaluate the MHT technique in order to find the best strategy for the internalization of MNPs coated with aminosilane in glioblastoma tumor cells. In this study, it was shown that the MHT technique was promising as an antitumor treatment, based on the intra- and extracellular effects of MHT. The optimization of the nanoparticle internalization process associated with its magnetic characteristics enhanced the acute extracellular and late intracellular effects of MHT, achieving greater efficiency in the therapeutic process. The publication by Żuk et al. [3] showed the potential of a multifunctional nanoparticle conjugate to act as a magnetic agent, anticancer immunological drug, and radiopharmaceutical in anticancer therapy, showing that radiobioconjugates have high potential for in vivo applications combining MHT therapy and immunotherapy against cancerous tissues. The study by Zhivago et al. [5] presented an alternative application of the MHT technique for the castration of animals, showing it to be effective in the castration of male rats in a short time, with few side effects on the general health of the animals.

The publication by Abbas et al. [2] developed carrier bioemulsomes with SPION-leflunomide for the intra-articular treatment of arthritis. This study concluded that these bioemulsomes can be considered an efficient platform for the suppression of rheumatoid arthritis.

Referring to studies using MNPs for diagnosis, the work by Nucci et al. [8] determined the ideal conditions for labeling mesenchymal stem cells used in cell therapy for stroke. In this study, MNPs coupled with fluorophores were used for in vivo detection using optical techniques. The findings in this study demonstrate the potential of multimodal MNPs for the theranostic evaluation of diseases. Akhma-deev et al. [7] developed nanoparticulate contrast agents as low-hemagglutinating-activity MNPs with applications in MRI. On the other hand, the publication by Friedrich et al. [6] presented the application of MNPs functionalized with peptides in the detection of Gram+ bacteria for the diagnosis and treatment of infectious diseases.

In the work of Li et al. [9] and Huang et al. [10], MNPs were addressed with a view to theranostic applications. Thus, Li et al. developed a polydopamine-based nanocarrier modified with MNPs and Pt nanoparticles loaded with necrostatin-1 (Nec-1), showing the great potential of this nanomaterial for the bimodal imaging and therapy of lupus nephritis. In the study by Huang et al., theranostic liposomes involving MNPs and low-dose therapeutic drugs conjugated with peptides were synthesized for the diagnosis and therapy of early atherosclerotic plaques in a preclinical model, showing high-potential theranostic effects.

The reviews by Gomez et al. [11] and Ahmad et al. [12] addressed the medical applications of metallic bismuth and lanthanide oxide nanoparticles, respectively. In the study by Gomez et al., the use of metallic bismuth nanoparticles for diagnostics in X-ray, photoacoustic, and fluorescence images was highlighted. In the therapeutic field, these metallic nanoparticles have shown potential as X-ray radiosensitizers for use in radiotherapy and as photothermal agents for applications in near-infrared phototherapy. In the review by Ahmad et al., functionalized lanthanide oxide nanoparticles were shown to have applications as contrast agents as well as applications in tumor targeting, medical imaging, and therapy.

In general, in this Special Issue, original articles and literature reviews are published that allow for understanding the importance of MNPs and potentiate their functionalization aiming at theranostic processes, giving future perspectives to this type of nanomaterial.
